# Hypoxia Promotes Neutrophil Survival After Acute Myocardial Infarction

**DOI:** 10.3389/fimmu.2022.726153

**Published:** 2022-02-11

**Authors:** Maximilian Dölling, Markus Eckstein, Jeeshan Singh, Christine Schauer, Janina Schoen, Xiaomei Shan, Aline Bozec, Jasmin Knopf, Georg Schett, Luis E. Muñoz, Martin Herrmann

**Affiliations:** ^1^ Department of Internal Medicine 3 - Rheumatology and Immunology, Friedrich-Alexander-University Erlangen-Nürnberg (FAU) and Universitätsklinikum Erlangen, Erlangen, Germany; ^2^ Deutsches Zentrum für Immuntherapie (DZI), Friedrich-Alexander-University Erlangen-Nürnberg and Universitätsklinikum Erlangen, Erlangen, Germany; ^3^ Department of Surgery, University Hospital Magdeburg, Magdeburg, Germany; ^4^ Institut für Pathologie, Universitätsklinikum Erlangen, Friedrich-Alexander-Universität Erlangen-Nürnberg, Erlangen, Germany

**Keywords:** neutrophils, acute myocardial infarction, hypoxia inducible factor 1, DNA decondensation, NET formation, neutrophil extracellular traps

## Abstract

Phagocytosis, degranulation, and neutrophil extracellular traps (NETs) formation build the armory of neutrophils for the first line of defense against invading pathogens. All these processes are modulated by the microenvironment including tonicity, pH and oxygen levels. Here we investigated the neutrophil infiltration in cardiac tissue autopsy samples of patients with acute myocardial infarction (AMI) and compared these with tissues from patients with sepsis, endocarditis, dermal inflammation, abscesses and diseases with prominent neutrophil infiltration. We observed many neutrophils infiltrating the heart muscle after myocardial infarction. Most of these had viable morphology and only few showed signs of nuclear de-condensation, a hallmark of early NET formation. The abundance of NETs was the lowest in acute myocardial infarction when compared to other examined diseases. Since cardiac oxygen supply is abruptly abrogated in acute myocardial infarction, we hypothesized that the resulting tissue hypoxia increased the longevity of the neutrophils. Indeed, the viable cells showed increased nuclear hypoxia inducible factor-1α (HIF-1α) content, and only neutrophils with low HIF-1α started the process of NET formation (chromatin de-condensation and nuclear swelling). Prolonged neutrophil survival, increased oxidative burst and reduced NETs formation were reproduced under low oxygen tensions and by HIF-1α stabilization *in vitro*. We conclude that nuclear HIF-1α is associated with prolonged neutrophil survival and enhanced oxidative stress in hypoxic areas of AMI.

## Introduction

Neutrophils are the first circulating cells to respond to microbial attacks or tissue damage. They initiate and orchestrate the inflammatory response (1). Besides performing phagocytosis and degranulation, neutrophils are capable to externalize chromatin and form Neutrophil Extracellular Traps (NETs) at sites of inflammation (2). The expelled chromatin is decorated with a plethora of nuclear and granular proteins; several of these display enzymatic activities. Initially NET formation is proinflammatory, however, at high neutrophil densities the NETs tend to aggregate and form aggregated NETs (aggNETs). The latter are endowed with robust proteolytic activities that degrade various soluble cytokine and toxic compounds in the resolution phase of inflammation (3–5).

During infection, NETs immobilize and kill bacteria, protozoa and fungi and limit their spread (6–10). Besides this beneficial, physiologic role, NETs and aggNETs reportedly also induce adverse effects. Indeed, NETs occlude Meibomian glands (11) and pancreatic ducts (12), promote formation and growth of gallstones (13) and salivary stones (14), and precipitate vasculopathy as well as immunothrombosis in COVID-19 (15). Furthermore, NETs may contribute to septic multi organ failure during tumor thrombosis (16–18) and malaria (19). Dysregulated NET metabolism and chronic persistence of circulating NETs and its degradation products, namely cell-free DNA, citrullinated Histone H3, Myeloperoxidase-/Neutrophil Elastase-DNA complexes, precipitate autoimmunity and inflammatory vessel damage (20–22).

After stimulation of the neutrophil, the NOX2-dependent oxidative burst and mitochondrial production of reactive oxygen species (ROS) are crucial in many pathways of NET formation (23). ROS initiate the disintegration of the nuclear envelope and the granular membranes (24). Although pathways independent of ROS have been described (10), conventional suicidal NET formation largely depends on the capability of the membrane-bound NADPH oxidase and myeloperoxidase (MPO) to produce hydrogen peroxide and its derivatives (25, 26). A sufficient supply with oxygen warrants the continuous formation of NETs in tissues (27). Slightly alkaline microenvironment favors the formation of NETs while acidic conditions are associated with reduced NET formation (28). Tissue hypoxia is often accompanied by a metabolic reprograming that conditions the acidification of the microenvironment. Interestingly, PMA induced NET formation was completely abolished in hypoxia but not NET formation triggered by Staphylococci (29). This indicates a fine regulation of the stimulating signals received by the neutrophil *in vitro*. Very little is known about the influence of tissue hypoxia on neutrophils *in vivo*.

Under normoxia, cytoplasmic hypoxia-inducible factor-1α (HIF-1α) is bound by hydroxyl groups and von Hippel- Lindau protein (VHL) and, consequently, degraded by the proteasome (30). In hypoxia, HIF-1α is protected from degradation by the prolyl hydroxylase domain-containing proteins (PHDs) and accumulates in the nucleus, where it induces the transcription of hypoxia-regulated genes. The most prominent transcripts are vascular endothelia growth factor (VEGF), erythropoietin (EPO) and adrenomedullin (31–33). In neutrophils, HIF-1α induces the transcription of NF-κB, which leads to reduced apoptosis and prolonged survival of neutrophils in hypoxia (34). The role of HIF-1α and hypoxia during NET formation *in vivo* has not been studied yet.

NET formation in normoxia or hypoxia setups was evaluated by (I) stimulation of isolated human peripheral blood neutrophils, and (II) collection of human tissue samples from acute myocardial infarction and other neutrophil-rich diseases without overt hypoxia through immunohistochemical analysis of HIF-1α and neutrophil elastase (NE). We sought to compare the level of expression of HIF-1α in apparently healthy neutrophils with neutrophils displaying already de-condensed nuclei on a single cell level in systematic immunofluorescence analyses of various human tissues.

Here we report that in acute myocardial infarction, a condition of pronounced hypoxia, most neutrophils showed high nuclear levels of HIF-1α and displayed a viable nuclear morphology. In contrast, the neutrophils that had started chromatin decondensation showed a low HIF-1α content. Furthermore, NET formation *in vitro* was reduced under hypoxia and HIF-1α stabilization by two PHDs inhibitors, roxadustat (RXD) and cobalt^2+^ (Co^2+^). These observations illustrate that hypoxia modulates NET formation *in vitro* and *in vivo* deepening our understanding of the influence of the microenvironment on the biology of neutrophils during initiation and resolution of sterile inflammation.

## Material and Methods

### Patients

Investigations on human material were performed in accordance with the Declaration of Helsinki and with the approval of the ethical committee of the University Hospital Erlangen (permit 243_15 B and permit 3755). A written informed consent was given by each donor.

### Neutrophil Isolation

Heparinized blood (20 U/ml) was drawn and diluted 1:1 with phosphate buffered saline (PBS) without calcium and magnesium (Thermo Fisher Scientific, Waltham, MS, US) and centrifuged at 350 RCF for 30 min at room temperature on a Ficoll (Bio-Rad, 824012, Dreieich, Germany) density gradient. The polymorphonuclear cells (PMNs) are separated from mononuclear cells forming a layer on the top of the red blood cells. PMNs were collected and subjected to 2 short cycles of hypotonic lysis with 36 ml deionized water. Osmolarity was restituted after 20 s with the addition of 4 ml 10 x PBS. Isolated PMNs containing more than 98% of neutrophils were suspended in PBS without calcium and magnesium and used for *in vitro* experiments immediately.

### Normoxia and Hypoxia

Buffer medium R_0_ consisting of RPMI 1640 containing 24 mM NaHCO_3_ (Gibco Thermo Fisher Scientific) and supplemented with 1 mM CaCl_2_ was equilibrated to pH 7.4 by incubation for 24 hours in a 5 % CO_2_ atmosphere. With the addition of 24 mM of NaHCO_3_ the pH was adjusted to 7.8. All buffers were equilibrated at 37°C, 5 % CO_2_ at 20 % oxygen for normoxia and at 2 % oxygen for hypoxia for 24 hours in a Heracell CO2 Incubator (ThermoFisher Scientific, Rockford, USA) or in the Whitley H35 hypoxystation (Meintrup-DWS, Bingley, UK) before starting the experiments, respectively.

### HIF-1α Stabilization

Equilibrated R_0_ medium was used to incubate the isolated neutrophils in R_0_ with 5µM Roxadustat for 30 min prior to the addition of the stimuli (RXD; Selleckchem, Houston, USA) or in the presence of cobalt chloride (Co^2+^, Sigma-Aldrich C8661, Taufkirchen, Germany) at the indicated concentrations during the stimulation period. RXD is an inhibitor of the prolyl hydroxylase domain-containing proteins (PHDs) which hydroxylates HIF-1α avoiding its ubiquitination and proteasome degradation (30). Co^2+^ oxidates ascorbate which is essential to reduce Fe^3+^ to Fe^2+^ in the catalytic sites of PHDs, thus inhibiting its activity and stabilizing HIF-1α in cells (35, 36).

### NET Assays

The sytox DNA externalization assay was employed to quantify the amount of DNA expelled by the neutrophils during NET-formation. Briefly, neutrophils (150,000/well) were seeded in 96-well plates (Greiner, flat bottom transparent polystyrene plate) in R_0_ buffer. The membrane impermeable DNA-dye sytox green was added (3.2 µM) just before starting the incubation at the indicated conditions. Extracellular trap formation was triggered by addition of LPS from *Klebsiella pneumoniae* at 2.5 µg/ml, PMA at 10 ng/ml, Ionomycin at 5µg/ml and 24 mM bicarbonate (pH 7.8) in R_0_ buffer medium. The increase of the sytox green fluorescence (ΔF) emitted at 525nm after 4 hours of incubation was recorded with the plate reader Infinite F200 Pro fluorometer (TECAN, Männedorf, Swiss). NET formation was confirmed by indirect immunofluorescence staining of neutrophils cultured on chamber slides under the same conditions. The percentage of inhibition of NET formation by various concentrations of Co^2+^ was calculated relative the ΔF of sytox green of those wells without Co^2+^.

### Indirect Immunofluorescence

Tissue samples from autopsies or biopsies were fixed in 4% formalin, dehydrated and embedded in paraffin blocks. Three μm sections were mounted and dried on Superfrost Plus slides (Thermo Fisher Scientific, Waltham, MS, US). Sections were dewaxed by incubation at 64°C for 1 hour. Paraffin residues were washed out in multiple steps with a solution containing decreasing concentrations of RotiHistol (Roth Chemie GmbH, Karlsruhe, Germany). Sections were rehydrated with Isopropanol following by incubation at 90°C for 20 min in Epitope Retrieval Solution (50 mM sodium citrate, pH 6). Chamber slides containing *in vitro* stimulated neutrophils were washed with PBS and fixed with 4% paraformaldehyde. Sections and chamber slides were then rinsed with deionized water, and permeabilized for 5 min with 0.5 % Triton X100 in PBS at RT.

The fixed slides were treated with blocking buffer (PBS supplemented with 1 % BSA; 2 % goat serum; 0.05 % Tween 20; 0.5 % Triton X100) for 2 h. Unconjugated primary antibodies (**Table 1**) were diluted in blocking buffer and incubated on the slides over night at 4°C. On the next day, the slides were washed with deionized water twice and rinsed with PBS once. Secondary antibodies (**Table 1**) were mixed with propidium iodide (PI, Sigma, P4641) for co-staining of DNA in blocking buffer. The mixture was added for 1.5 h at RT in darkness. Slides were rinsed three times with PBS, and embedded in DAKO fluorescent mounting medium (BIOZOL, Eching, Germany). Analysis was performed employing a BZ-X710 fluorescence microscope (Keyence, Neu-Isenburg, Germany) and (Nikon GmbH, Düsseldorf, Germany). We conducted Z-stacks in order to increase depth and to improve the visualization of NETs on these images. Images were post-processed using Photoshop CS6 (Adobe, München, Germany).

**Table 1:** Antibodies used in immune fluorescence.

	Serial number	Company
**Primary antibodies**		
Rabbit anti-human neutrophil elastase; 1:100	ab68672	Abcam, United Kingdom
Rabbit anti-human anti-HIF-1α	Orb378857	Biorbyt, Cambridge, United Kingdom
1:100		
**Secondary antibodies**		
Cy5-conjungated goat anti-rabbit IgG; 1:400	111-175-144	Jackson ImmunoResearch, Suffolk, United Kingdom
AF647-Goat anti-Rabbit IgG (H+L)	ab150079	Abcam, United Kingdom
Invitrogen (1:400)		

### Apoptosis Detection by Flow Cytometry

For the quantification of cell viability and apoptosis, neutrophils incubated under normoxia and hypoxic conditions for 4 hours were stained with annexin A5 (AxA5-FITC) and propidium iodide (PI). In brief, 100 µl of cell suspension was incubated for 30 min at 4°C with 400 µl Ringer`s solution (Braun) containing 0.5 µg/ml AxA5-FITC 2 µg/ml PI (Sigma-Aldrich). Then, cells were analyzed with a Gallios™ flow cytometer (Beckman Coulter, Hialeah, FL). FITC fluorescence was excited with 488 nm and recorded on a fluorescence 1 (FL1) channel (525 nm BP 40). PI fluorescence was excited with 488 nm and recorded on a fluorescence 3 (FL3) channel (620 nm BP 30). Data were processed with Kaluza™ software, version 2.1, after electronic compensation to eliminate bleed through of fluorescence. At least three independent experiments, each in triplicates, were performed. Acquired neutrophils were classified in viable (AxA5/PI negative), apoptotic (AxA5 positive/PI negative), and necrotic (AxA5 positive/PI positive) cells, respectively.

### Oxygen Saturation of Culture Medium in a Cell-Free System

In order to determine the reduction of oxygen saturation of medium, we employed 96-well microplates Oxoplates^®^ with integrated chemical optical oxygen sensors (Pressens, Regensburg, Germany). Oxygen saturated medium with and without 100µM ascorbate (Sigma-Aldrich, A92902, Taufkirchen, Germany) was incubated with the indicated concentrations of Co^2+^ for 1 hour. The percentage of oxygen saturation was calculated according manufacturer instructions after measuring the fluorescence from two filter pairs for the indicator (excitation: 535 nm, emission: 680 nm) and reference dye (excitation: 540 nm, emission: 590 nm).

### Quantification of Reactive Oxygen Species by Flow Cytometry

For the measurement of intracellular ROS, isolated neutrophils were loaded for 20 minutes with 2 µM CM-H2DCFDA (ThermoFisher) at in PBS. For the measurement of mitochondrial ROS, isolated neutrophils were loaded for 10 minutes with 5 µM MitoSOX™ (ThermoFisher) in PBS. The ROS produced by the neutrophils was calculated as the fold increase of the fluorescence detected by the FL1 (525 nm BP 40) and FL2 (575 BP 30) channels of the Gallios Flow Cytometer upon stimulation with 100 ng/ml PMA for 30 minutes.

### Cytomorphometry

Distribution and co-localization of fluorescence signals of immunofluorescence images were processed using self-generated macros for Photoshop CS6. Staining, magnification, exposure, and processing were identically performed for all samples. Positive samples and negative controls were simultaneously co-processed. Regions infiltrated with neutrophils (neutrophil elastase) of various pathologies were selected and the grey levels of Cy5 and PI were recorded. Signals with a grey level less than 4 were considered remaining background signal and not included in the analysis. Neutrophil elastase positive areas were classified according to its appearance as either condensed multilobulated neutrophils (viable neutrophils) or as diffuse extracellular NE pattern (NET aggregates). The ratio of the area of the NE-signal associated with lobulated nuclei to the total area of the elastase signal defined the % area of neutrophil elastase in viable neutrophils. The ratio of the area of the NE-signal associated with NET aggregates to the total area of the elastase signal defined the % area of neutrophil elastase of NET aggregates. Various forms of neutrophils and NETs were observed and are described and summarized in the **Table 2**. The HIF-1α fluorescence in myocardial tissues was also quantified recording the grey value (MFI) of the pixels co-localizing with PI fluorescence. The nuclear area of each leukocyte was quantified by the pixel area of PI fluorescence. A Pearson correlation analysis between the HIF-1α fluorescence intensity and the nuclear area of the leukocyte infiltrates was performed.

**Table 2:** Morphological forms of tissue neutrophils and NETs.

Morphologic form	Features
Viable neutrophils	Many granules with 0.0025-0.25 µm²; granular cytoplasmic elastase not-colocalizing with nuclear chromatin; no or inactive cytoplasmic HIF-1α; 90-100 µm²
Degranulated neutrophils	Few granules; granular cytoplasmic elastase not-colocalizing with nuclear chromatin; no or inactive cytoplasmic HIF-1α; 90-100 µm²
Granules	High levels of elastase; no DNA; 0.0025-0.25 µm²
Neutrophils with decondensed chromatin in hypoxic areas	Low active nuclear HIF-1α; 100-200 µm²
Viable neutrophils in hypoxic areas	High active nuclear HIF-1α; 90-100 µm²
NETs	Granular cytoplasmic elastase colocalizing with extranuclear chromatin; 400-1000 µm²
NET aggregates	Granular cytoplasmic elastase colocalizing with extranuclear chromatin; can extend almost unlimited

### Data Presentation and Statistical Analysis

Quantitative results are displayed as means and individual values. If not indicated otherwise, two-way analyses of variance (ANOVAs) were used for establishing statistical differences among groups. In case of multiple comparisons, either Sidak’s or Tukey´s multiple comparisons tests were applied. Statistical analysis was performed with the software GraphPad Prism 6.0 (GraphPad Software, USA).

## Results

### Morphologies of Tissue Neutrophils

**Figure 1:**
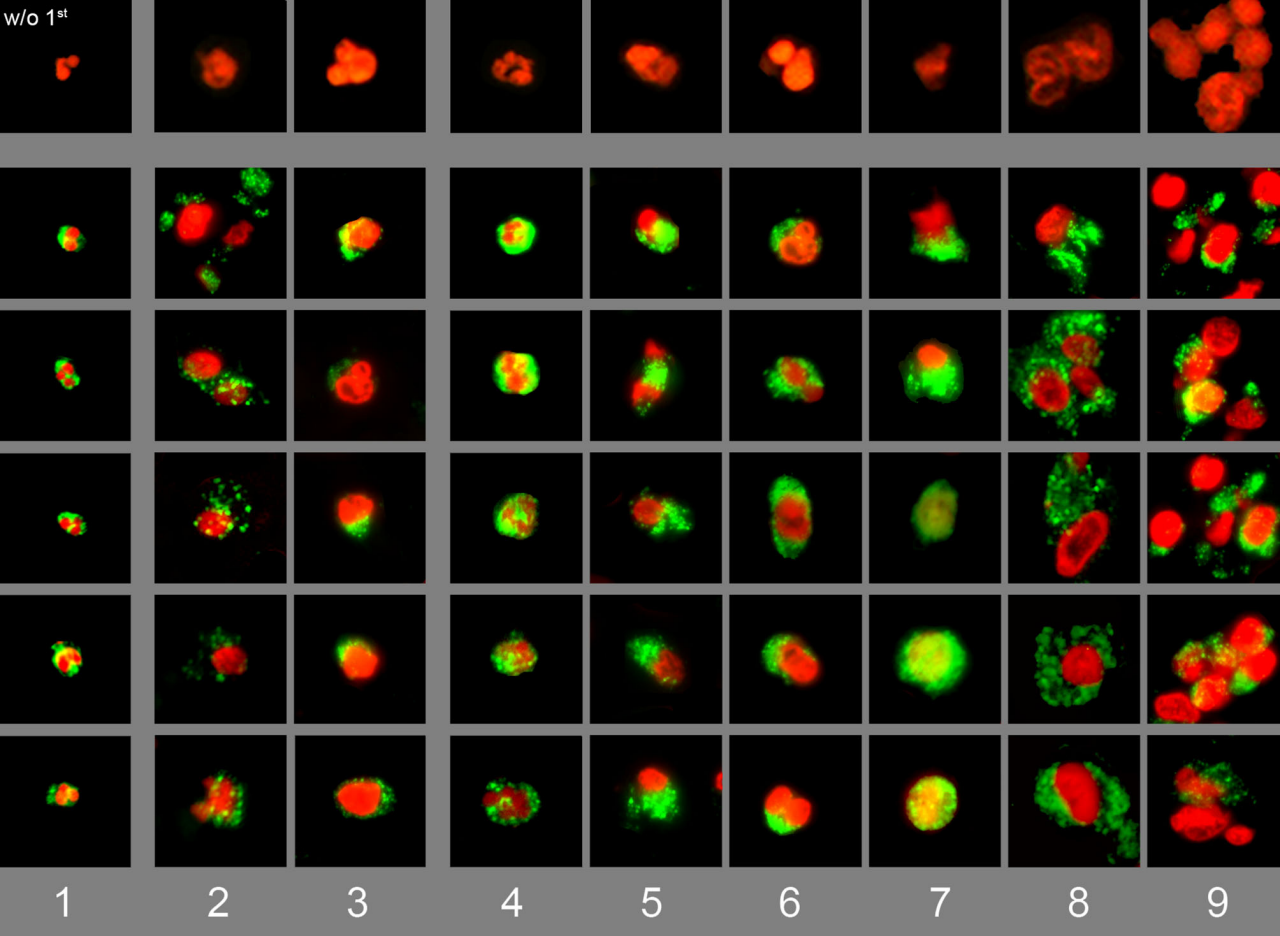
Composite image of various stages of degranulation or NET formation of tissue neutrophils. Immunofluorescence staining of various biopsies for DNA with PI (red) and for neutrophil elastase (green). Various types of tissue neutrophils were identified and classified in a composite image showing (1) viable neutrophils, (2) chromatin decondensation and spreading of granular content, (3) highly decondensed chromatin with associated granular remnants. (4-9) various steps of chromatin externalization: (4) nuclear swelling and increase of cellular diameter, (5-6) loss of nuclear shape, (7) mixture of DNA with granular enzymes, and (8-9) aggregation of swollen neutrophils with and without chromatin externalization. The upper panel shows selected nuclei from slides lacking of the first antibody. Each individual field represent 50 x 50 µm.

We stained tissue sections from various diseases for DNA with PI (red) and for neutrophil elastase (green). The most representative nuclei that were associated with neutrophil elastase signals were selected for a composite image showing various forms of appearance of neutrophil in tissues (**Figure 1**). The specificity of the anti-neutrophil elastase antibody was confirmed in non-infiltrated areas of the same section (**Supplementary Figure 1**). The nuclei of viable neutrophils showed a lobulated morphology with cytoplasmic neutrophil elastase (**Figure 1**, column 1). Some neutrophils had swollen nuclei and showed extracellular spreading of neutrophil elastase (signs of degranulation). In some cases neutrophil elastase remnants were found associated to the nuclear membrane (columns 2-3). Various steps of chromatin externalization of NET forming neutrophils were observed (columns 4-9): DNA decondensation, nuclear swelling and increase of cellular diameter (4), deformation of the nucleus (5, 6), and mixture of chromatin with granular enzymes (7). Finally, spreading of chromatin and aggregation of neutrophils without NETs (8) or in aggregated NETs (9). Interestingly, the predominant morphologic form of neutrophils varied among the investigated diseases. The main morphological features of tissue neutrophils are summarized in **Table 2**.

**Figure 2:**
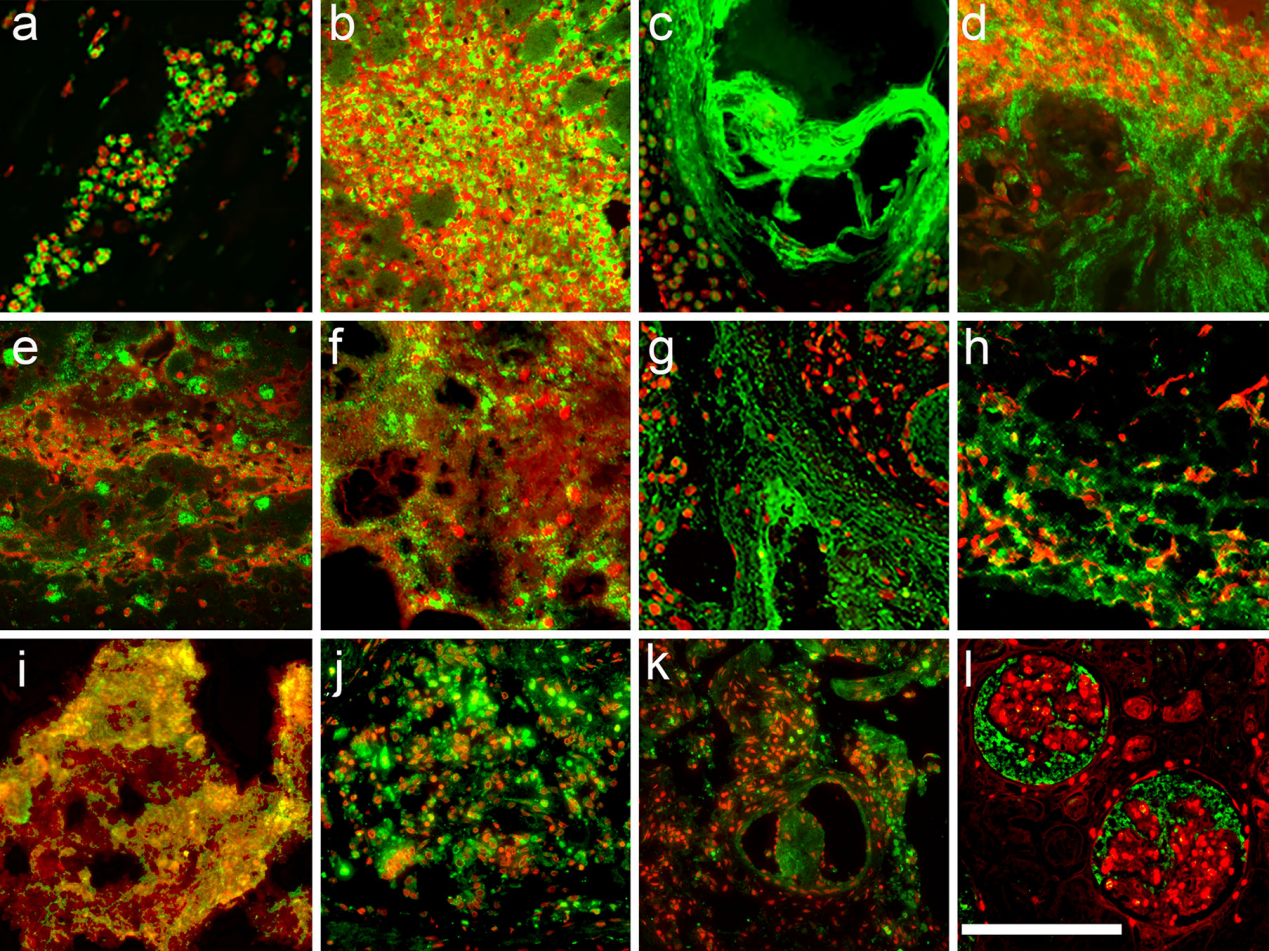
Viable neutrophils dominate samples from acute myocardial infarction. Immunofluorescence microphotographs of histological sections of various pathologies stained for neutrophil elastase (green) and counterstained for DNA (PI; red). Note, in acute myocardial infarction **(A)** neutrophils showed lobulated nuclei, cytoplasmic granular elastase and little extracellular DNA, indicating viable neutrophils. In contrast, in hepatic abscess **(B)**, inflamed dermal tissue in systemic lupus erythematodes **(C)**, acute pancreatitis **(D)**, infectious endocarditis **(E, F)**, Sjögren syndrome **(G)**, abdominal aortic aneurysm **(H)**, infiltrated dental calculi **(I)**, sinus pilonidalis abscess **(J)**, vascular plugs in the lungs of COVID-19 **(K)**, and glomerulonephritis in COVID-19 **(L)** most neutrophils have formed NETs or NET aggregates. The size bar represents 200 µm.

A total of 145 tissue samples of 67 patients with various diseases (**Table 3**) were stained for DNA (PI) and neutrophil elastase. In acute myocardial infarction, a disease with tissue damage caused by severe hypoxia, the DNA of most neutrophils (PI; red) displayed a condensed multilobulated nuclear appearance and neutrophil elastase (immune fluorescence; green) was invariably found in the cytoplasmic granules (**Figure 2A**). We consider these cells viable neutrophils. In contrast, in abscesses (**Figures 2B, J**), acute pancreatitis (**Figure 2D**), infectious endocarditis (**Figures 2E, F**), COVID-19 (**Figure 2K**) and many more conditions (**Figures 2B**–**L**; **Table 3**) the neutrophil elastase appeared often diffuse and associated or not with extracellular chromatin/DNA. This suggests abundant degranulation and/or formation of neutrophil extracellular traps.

**Table 3:** Neutrophil-driven diseases included in this study.

Pathology	Number of sections	Number of patients
Acute myocardial infarction	25	7
Infectious endocarditis	23	5
Abscesses*	22	6
Thrombosis in COVID-19 infection**	22	6
Acute pancreatitis	20	4
Dermal inflammation***	12	12
Infiltrated dental calculin	14	14
Granuloma	6	3
Abdominal aortic aneurysm	5	5
Sjögren Syndrome	4	4
Liver failure in COVID 19	4	1
**Total**	**145**	**67**

*Several abscesses were included from different sites of the body: dermal abscess (1), liver abscess (1), perianal abscess (1), perimandibular abscess (1), pharyngeal abscess (1), and sinus pilonidalis abscess (1).

**Thrombosis in COVID-19 patients were found in central lungs (6), peripheral lungs (9) and in the kidneys (7).

***Dermal inflammation included sections from following pathologies: pemphigoid (2), psoriasis pustulosa (4), and systemic lupus erythematodes (6).

**Figure 3:**
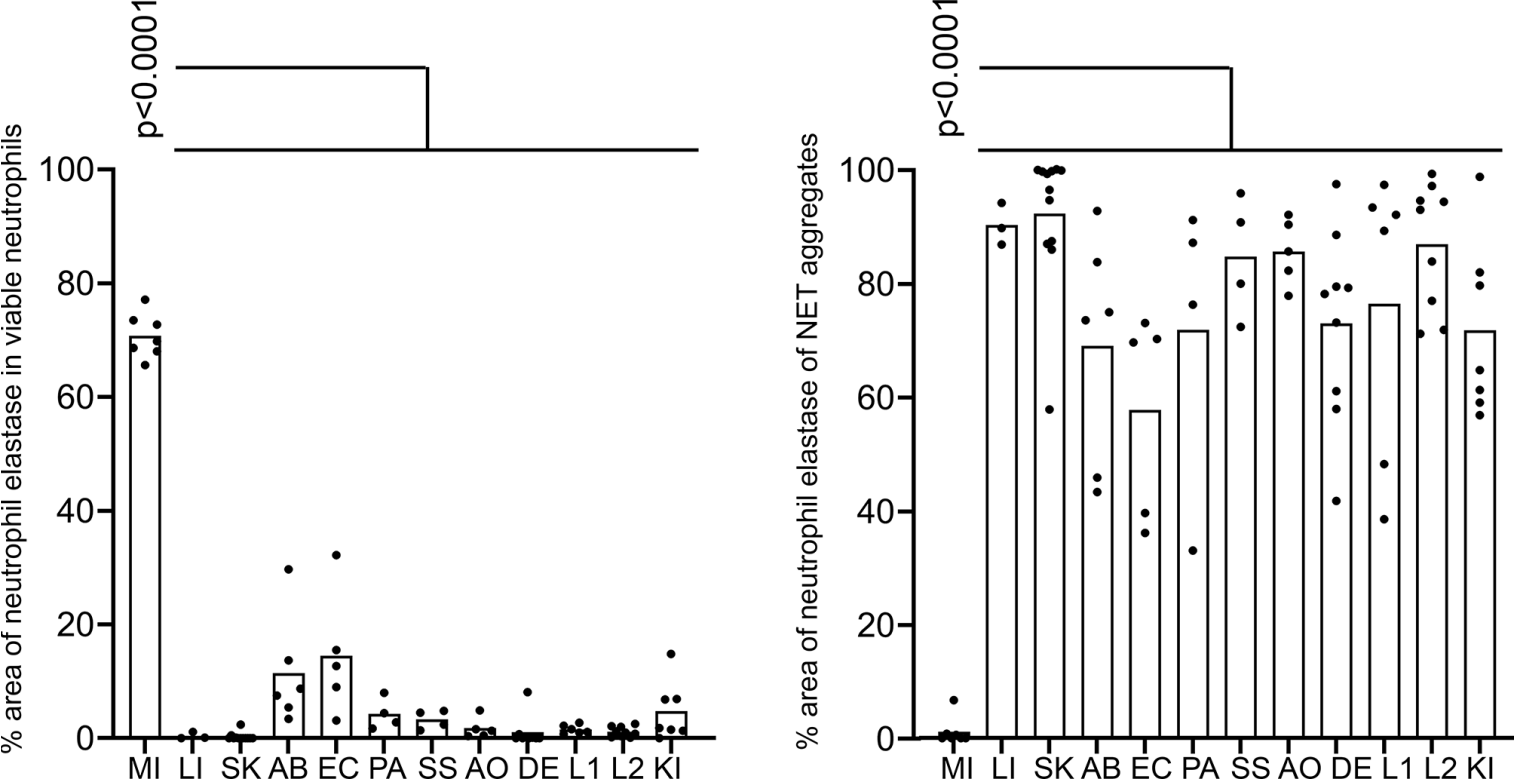
Pattern of neutrophil elastase in various tissues. Cytomorphometry of viable neutrophils and NETs (aggregates) found in tissue sections described in **Table 1** and exemplarily shown in **Figure 2**: MI, acute myocardial infarction; LI, liver of a patient with severe COVID-19; SK, epidermal inflammation; AB, abscesses; EC, heart muscle of patients with infective endocarditis; PA, pancreatitis; SS, lip salivary glands of patients with Sjögren’s syndrome; AO neutrophil infiltrates of patients with aortic aneurysms; DE, dental calculi infiltrated by neutrophils; L1, vascular plugs in the central lung of patients with COVID-19; L2, vascular plugs in the peripheral lung of patients with COVID-19; KI, renal thrombosis in patients with COVID-19. Note that in acute myocardial infarction tissue neutrophils remained predominantly viable compared to other diseases (p < 0.0001). Conversely, neutrophils formed large amounts of aggregates in all other diseases, but not in acute myocardial infarction (p < 0.0001). The p values are from a two-way ANOVA after Tukey´s multiple testing correction.

The cytomorphometric analyses showed that viable neutrophils with cytoplasmic pattern of neutrophil elastase immunostaining dominated the sections of patients with acute myocardial infarction (**Figure 3**). Whereas only few viable neutrophils were to be detected in the other diseases (p < 0.0001). In all other pathologies analyzed mostly diffuse neutrophil elastase immunostaining associated or not with extracellular chromatin/DNA was observed (p < 0.0001).

### NET Formation Under Hypoxia and Stabilized HIF-1α

**Figure 4:**
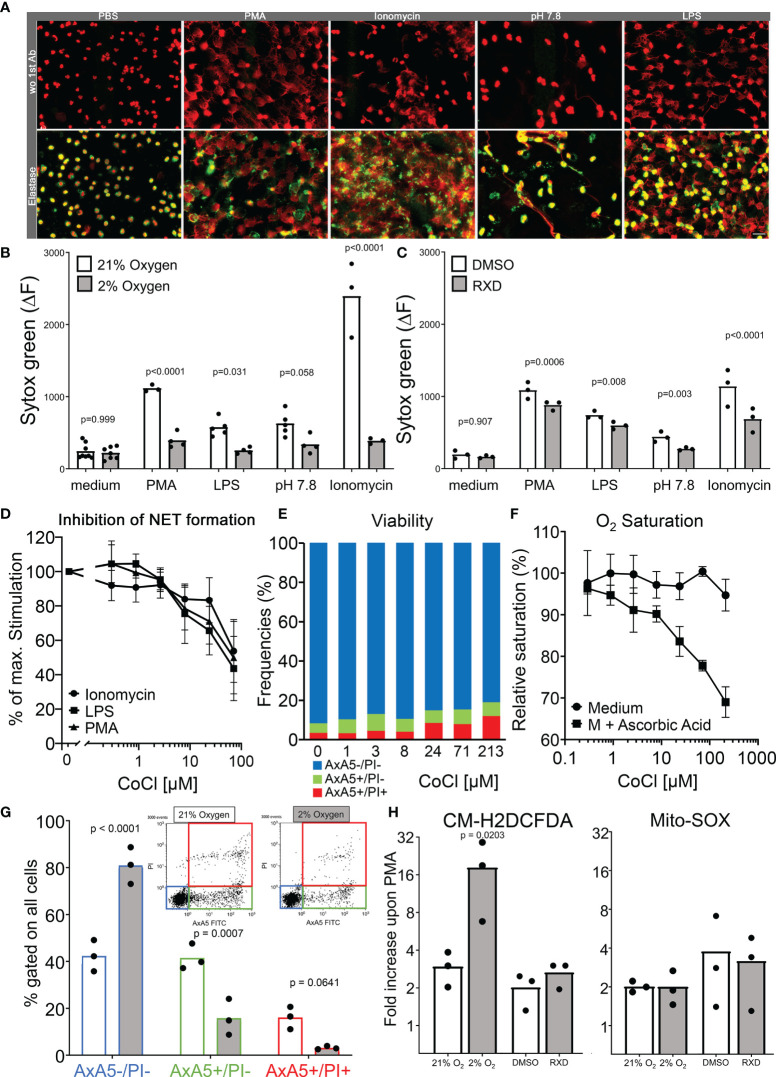
Modulation of neutrophil functions during hypoxia. Immunofluorescence stainings of stimulated neutrophils in chamber slides showing nuclear decondensation, DNA spreading and colocalization of neutrophil elastase with extracellular DNA **(A)**. The quantification of NET formation in hypoxia and normoxia was done by DNA externalization. The delta of the Sytox green fluorescence between 0 and 4 hours of incubation in normoxia and hypoxia is shown in **(B)** and after HIF-1α stabilization (RXD) in **(C)**. Doses-dependent inhibition of NET formation by Co^2+^ in sytox assays after stimulation with Ionomycin, LPS and PMA **(D)**. Frequencies of viable (AxA5-/PI-), apoptotic (AxA5+/PI-), and necrotic (AxA5+/PI+) neutrophils after 4 hours incubation in medium with increasing concentrations of Co^2+^ **(E)**. The oxygen saturation of medium in the presence of increasing concentrations of Co^2+^ after the oxidation of ascorbic acid for 1 hour **(F)**. The proportion of viable (AxA5-/PI-), apoptotic (AxA5+/PI-), and necrotic (AxA5+/PI+) neutrophils after 4 hours incubation in normoxia and hypoxia are shown in **(G)**. The production of ROS in the cytoplasma (CM-H2DCFDA) and mitochondria (MitoSOX) of neutrophils was measured upon PMA stimulation under hypoxia and HIF-1α stabilization. Displayed are the fold increase of the fluorescence of the indicated ROS sensors upon PMN stimulation after 30 minutes incubation **(H)**. Results were obtained from at least 3 independent experiments with 3 to 4 different blood donors. The p values are from a two way ANOVA after Sidak´s multiple testing correction.

We aimed to quantify NET formation triggered by ionomycin, PMA, LPS and pH 7.8 in hypoxia and normoxia (2 % and 21 % O_2_, respectively). We confirmed in the first instance that neutrophils treated with the mentioned stimuli formed NETs. Spread extracellular DNA were decorated with neutrophil elastase identified as NETs in immunostainings of neutrophil cultures (**Figure 4A**). In order to quantify changes in the ability to form NETs, a sytox green-based DNA-externalization assay was employed. In normoxia, PMA, LPS, elevated pH as well as ionomycin induced robust DNA externalization when compared to the unstimulated condition (p = 0.0093, p = 0.0205, p = 0.0116 and p = 0.0514 in the ANOVA corrected by Tukey´s multiple comparisons, respectively). Under conditions of hypoxia DNA externalization was partially abrogated. Neither PMA (p = 0.302), LPS (p = 0.838), elevated pH (p = 0.441) nor ionomycin (p = 0.097) induced significant DNA externalization when compared to the unstimulated condition. Furthermore, the intensity of DNA externalization induced under hypoxia was significantly lower than those measured under normoxic conditions (**Figure 4B**). We further analyzed induced DNA externalization after treatment with RXD, an inhibitor of PHDs, which hydroxylates HIF-1α avoiding its ubiquitination and proteasome degradation. The stabilization of HIF-1α led to a partial but significant reduction of DNA release after simulation with PMA, LPS, pH 7.8 and ionomycin (**Figure 4C**). We also stabilized HIF-1α by Co^2+^ which catalyzes the oxidation of ascorbate, favouring the Fe^3+^ redox state, inactivating PHD activity and stabilizing HIF-1α (35, 36). A doses-dependent inhibition by Co^2+^ of NET formation was observed in the DNA-externalization experiments (**Figure 4D**) without significant reduction of the viability of neutrophils (**Figure 4E**). Interestingly, the reduction of the oxygen saturation induced by the Co^2+^-dependent oxidation of ascorbate in the cell-free assays fairly overlaps with the inhibition of NET formation (**Figure 4F**). Additionally, we confirmed that under hypoxic conditions neutrophils are rescued from the spontaneous apoptosis and that most of the neutrophils retained their membrane integrity during the indicated incubation times (**Figure 4G**). Notably, hypoxia not only prolonged the lifespan of neutrophils *in vitro*, it also fostered the production of ROS in the cytoplasma without affecting the oxidative burst in the mitochondria of isolated neutrophils as measured by the probes CM-H2DCFDA and MitoSOX (**Figure 4H**).

**Figure 5:**
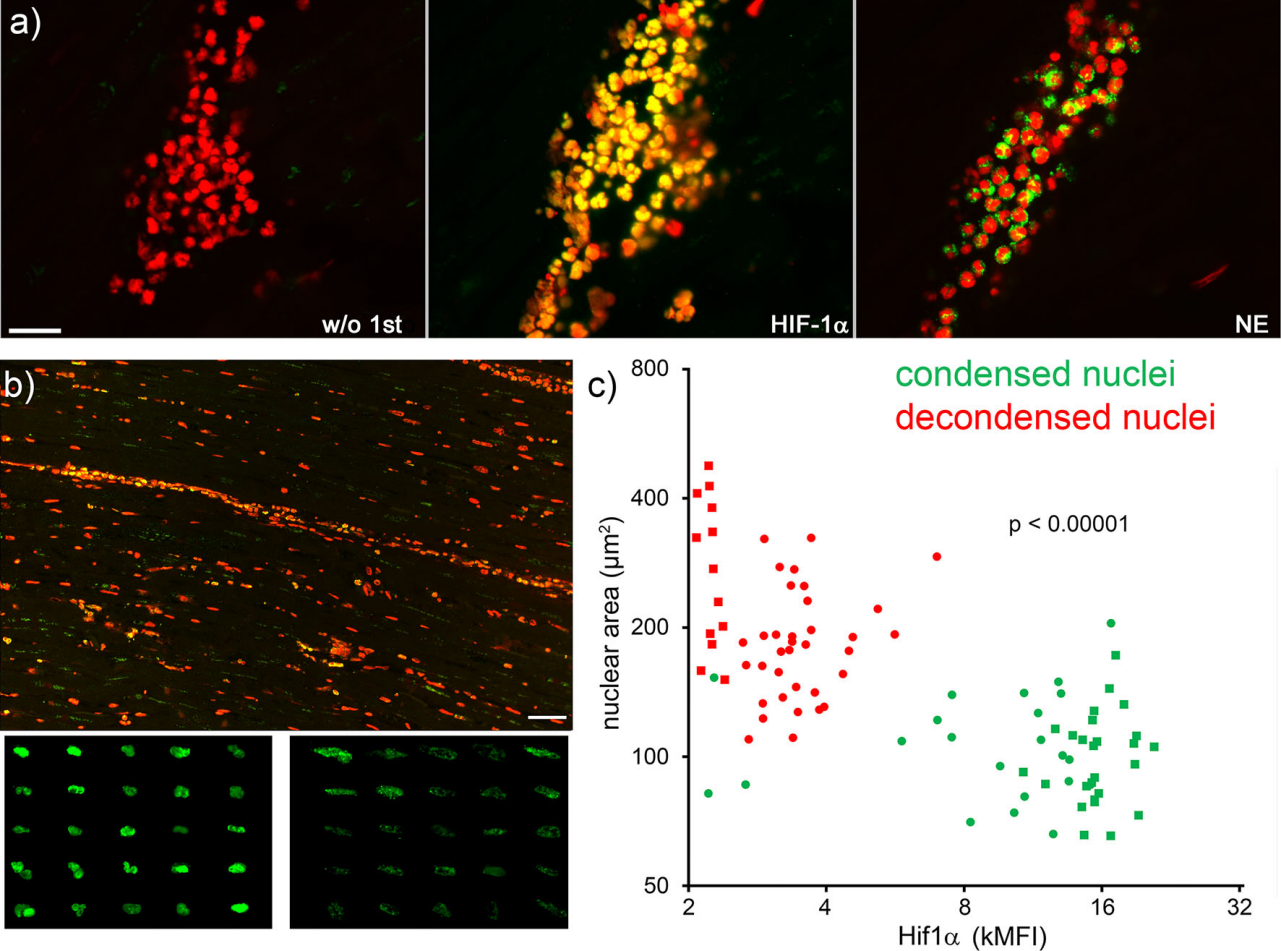
Neutrophils with high nuclear levels of HIF-1α under hypoxia display viable morphologies. Serial slices of neutrophil infiltrates of ischemic areas of human acute myocardial infarction were stained by immunofluorescence for HIF-1α (green), neutrophil elastase (NE, green) and DNA (PI, red) **(A)**. Overview (**B**, upper panel) and composite details (B, lower panels) of HIF-1α positive nuclei. The size bar represents 400 µm. Neutrophils with condensed nuclei (viable) showed bright HIF-1α staining (**B**, left lower panel), whereas neutrophils with decondensed nuclei (early stage of NET formation) contained less HIF-1α (**B**, right lower panel). The pattern and intensity of HIF-1α inversely correlated (*Pearson r* -0.5574, p <0.0001) with the nuclear area of neutrophils **(C)**. Round and square dots distinguish the sample of AMI analyzed (two samples). MFI, mean fluorescence intensity.

Next, serial tissue sections of patients with acute myocardial infarction were immunostained for nuclear HIF-1α, neutrophil elastase and DNA (**Figure 5A**). The cytomorphometry analysis showed that neutrophils with viable appearance contained more nuclear HIF-1α than those with decondensed nuclei (**Figures 5B, C**; p < 0.0001). The correlation analysis after Pearson showed a significant inverse correlation between the intensity of HIF-1α and the nuclear area of myocardial neutrophils (*r* -0.5574, p <0.0001).

## Discussion

The main function of NETs is to form surrogate barriers against invading pathogens during acute phase of inflammation (8). Additionally, NETs are endowed with potent intrinsic anti-microbial and anti-inflammatory activities that result vital for tissue homeostasis (3). The formation of NETs suppose the destruction of the neutrophil and the loss of other important defensive functions like degranulation and phagocytosis. Our systematic observational study of various pathologies demonstrates that NETs are found in tissues without overt ischemia and are practically absent in the ischemic myocardium. We sought therefore to evaluate the factors contributing to the decision of the neutrophil to form NETs based on the pattern of appearance of neutrophils, NETs and HIF-1α, the key factor in cell response to hypoxia, in human tissues.

Under hypoxia, HIF-1α is stabilized and transferred into the nucleus of neutrophils. This results in an increase of NF-κB, which mediates neutrophil survival and cytokine transcription (34). Our *in vitro* data indicate that low oxygen tension and nuclear HIF-1α strongly reduce the capacity of neutrophils to form NETs, prolong survival and favor the production of cytoplasmic ROS. This is in line with previous investigations showing that elevated expression of HIF-1α in neutrophils promotes their phagocytotic capabilities and prolongs their survival (37).

We confirmed in the human AMI that neutrophils displaying a viable multilobulated nuclei express high levels of HIF-1α in their nuclei. Conversely, low levels of nuclear HIF-1α were associated with DNA decondensation, an early step NET formation. Under hypoxic conditions, neutrophils exhibit elevated release of matrix metalloproteinase-9, lactoferrin, myeloperoxidase, and elastase suggesting increased degranulation and enhanced tissue damage potential (38). Branitzki-Heinemann et al. failed detecting up-regulation of the expression of HIF-1α in neutrophils exposed to hypoxia (29). HIF-1α nuclear translocation is reportedly required to maintain degranulation and chemotaxis active in neutrophils during hypoxia (39) while cytoplasmic HIF-1α is required for LPS induced NET formation (40). We see a clear nuclear accumulation of HIF-1α in dense multilobulated neutrophil nuclei *in vivo* and inhibition of NET formation at low oxygen tension and with the chemical stabilization of HIF-1α *in vitro*.

The chemical stabilization of HIF-1α simulates transcriptional responses of cells exposed to low oxygen tensions. Oxygen, ascorbate and 2-oxoglutarate are required to maintain Fe^2+^ in PHDs in a chain of well-balanced redox reactions (41). The amount of Co^2+^ required for the oxidation of ascorbate is directly proportional to the lack of oxygen. This assumption allows us to extrapolate the level of hypoxia required to inhibit NET formation by measuring the amount of oxygen consumed in the medium during the oxidation of ascorbate by Co^2+^. In this fashion, we observed a reduction of 20% of the NET formation by Co^2+^ with the same concentration of Co^2+^ that caused 20% reduction of the oxygen saturation of the medium. In terms of oxygen tension in the medium this would be around 120 mmHg pO_2_. Further experiments are needed to confirm this approximation.

Our observations along with the published data suggest specific roles of HIF-1α according to its subcellular localization in neutrophils. The main limitation of our observational study is the natural bias derived from unilateral observations. However, we have demonstrated with standard research tools and employing appropriate controls that neutrophils have different appearances in different human tissues and diseases. The molecular mechanism behind this particular behavior of neutrophils need further investigations in both, *in vitro* and *in vivo* models of ischemic disease.

In our experiments, the ability to produce ROS in the mitochondria of neutrophils was not affected by low oxygen tensions. Cytoplasmic oxidative burst was contrarily enhanced under hypoxia. This means that atmospheric oxygen is dispensable for the oxidative metabolism of the neutrophil and suggest that compensatory metabolic pathways are engaged to maintain neutrophil functions active at sites with low oxygen availability. It has been reported that the stabilization of HIF-1α up-regulate the pentose phosphate pathway ensuring the cytoplasmic supply of NADPH (42–44). Furthermore, the pharmacological activation of the glycolytic pathway through the phosphofructokinase-1 liver type, as it occurs during hypoxia, inhibits NET formation (45). In sum, low oxygen tensions prompt neutrophils to cause tissue damage through a sustained degranulation and secretion of reactive oxygen species and a reduction of pro-resolving NETs.

The decision between neutrophil survival and NET formation is also influenced by the equilibrium among CO_2_, bicarbonate and pH. NETs are readily externalized under conditions with high pH and a high ratio between ${\text{HCO}}_{3}^{-}$ and CO_2_, less NETs are formed under low pH (28). During ischemia, oxygen levels are diminished and the environmental pH is decreased (46). In myocardial infarction neutrophils are attracted by cell debris and inflammatory signals released by activated neighboring cells and massively infiltrate the infarcted area in the first few hours following onset of ischemia (47). Our observations on AMI demonstrate that neutrophils do not externalize chromatin at sites of ischemia and hypothesize that they remain active being able to remove cell debris or releasing matrix-degrading enzymes and reactive oxygen species. This concurs in a weakening of myocardial extracellular matrix and enhancement of the infarct area (48). Elevated rates of complications after AMI such as ventricular wall rupture or papillary muscle disruption are associated with neutrophil infiltration (49), indicating a pivotal role of neutrophils in wound healing and scar formation (50). Since the aggregation of NETs have been reported to degrade inflammatory mediators (3) and sequester proteolytic enzymes (51), we suggest that the lack of NET formation contributes the prolonged inflammatory insult to the myocardium in AMI.

## Author’s Note

The present work was performed in fulfillment of the requirements for obtaining the degree, Dr. med.” Maximilian Dölling.

## Data Availability Statement

The original contributions presented in the study are included in the article/**Supplementary Material**. Further inquiries can be directed to the corresponding author.

## Ethics Statement

Investigations on human material were performed in accordance with the Declaration of Helsinki and with the approval of the ethical committee of the University Hospital Erlangen (permit 243_15 B and permit 3755). A written informed consent was given by each donor. The patients/participants provided their written informed consent to participate in this study.

## Author Contributions

MD and ME conducted autopsies, performed general pathological analysis, coordinated patient sample acquisition and acquired ethical approval of the study. MD, JSc, XS and JSi performed immunofluorescence staining and performed tissue neutrophil studies. MD and MH processed the images. JSi, XS, JSc and CS performed *in vitro* hypoxia experiments. JK, AB, CS, LM and MH supervised experiments and made data and statistical analyses. LM, AB, GS and MH conceived the study. All authors provided scientific input, wrote, read and approved the manuscript.

## Funding

This work was partially supported by the German Research Foundation (DFG) 2886 PANDORA Project-No.B3; SCHA 2040/1-1; CRC1181(C03); TRR241(B04), by the EU ERC-Synergy grant 4D Nanoscope and by H2020-FETOPEN-2018-2019-2020-01; 861878„NeutroCure”, and by the Volkswagen-Stiftung (Grant 97744).

## Conflict of Interest

The authors declare that the research was conducted in the absence of any commercial or financial relationships that could be construed as a potential conflict of interest.

## Publisher’s Note

All claims expressed in this article are solely those of the authors and do not necessarily represent those of their affiliated organizations, or those of the publisher, the editors and the reviewers. Any product that may be evaluated in this article, or claim that may be made by its manufacturer, is not guaranteed or endorsed by the publisher.
