# Supplementary material for: Hypoxia Promotes Neutrophil Survival After Acute Myocardial Infarction
Source: Front Immunol. 2022 Feb 11;13:726153. doi: 10.3389/fimmu.2022.726153 (PMC8873092; doi:10.3389/fimmu.2022.726153)

Supplementary Figure.

Validation of the antibody rabbit anti-human neutrophil elastase (Abcam ab68672) at 1:100 dilution.

Immunofluorescence of neutrophil elastase (green) counterstained with propidium iodide (DNA, red). Shown are formalin-fixed paraffin-embedded human tissues with neutrophil-infiltrated areas (right panel) and non-infiltrated areas of the same tissue slice. Scale bar 50 µm. Heart, acute myocardial infarction; Kidney, glomerulonephritis in COVID-19; Lung, COVID-19.

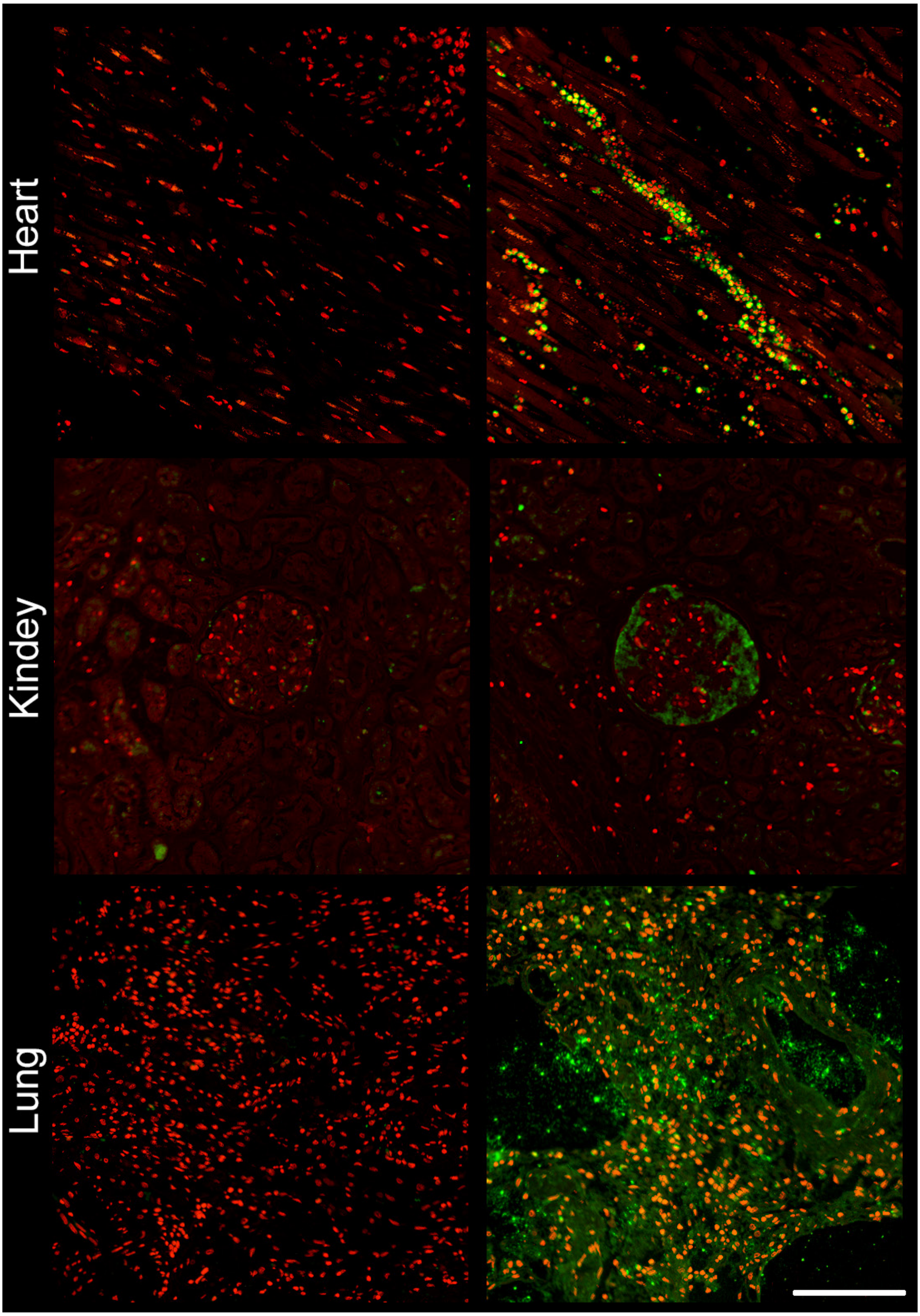

Supplement: Supplementary file 1 [file Image_1.pdf]
